# Supplementary figures and images for: LRRC8A Is a Promising Prognostic Biomarker and Therapeutic Target for Pancreatic Adenocarcinoma
Source: Cancers (Basel). 2022 Nov 10;14(22):5526. doi: 10.3390/cancers14225526 (PMC9688930; doi:10.3390/cancers14225526)

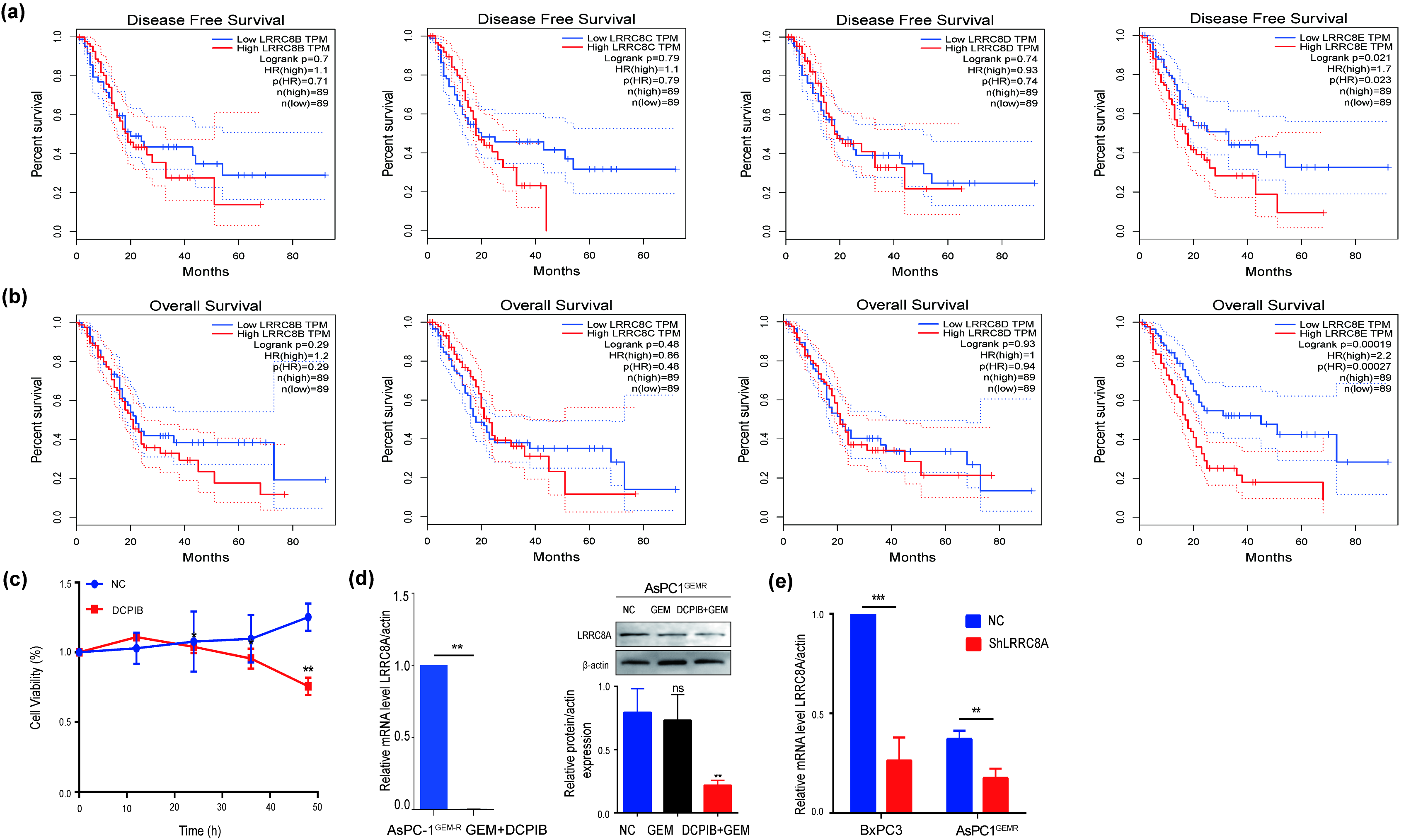

Supplement: Supplementary file 1 [file cancers-14-05526-s001.zip › Figure S1.tif]

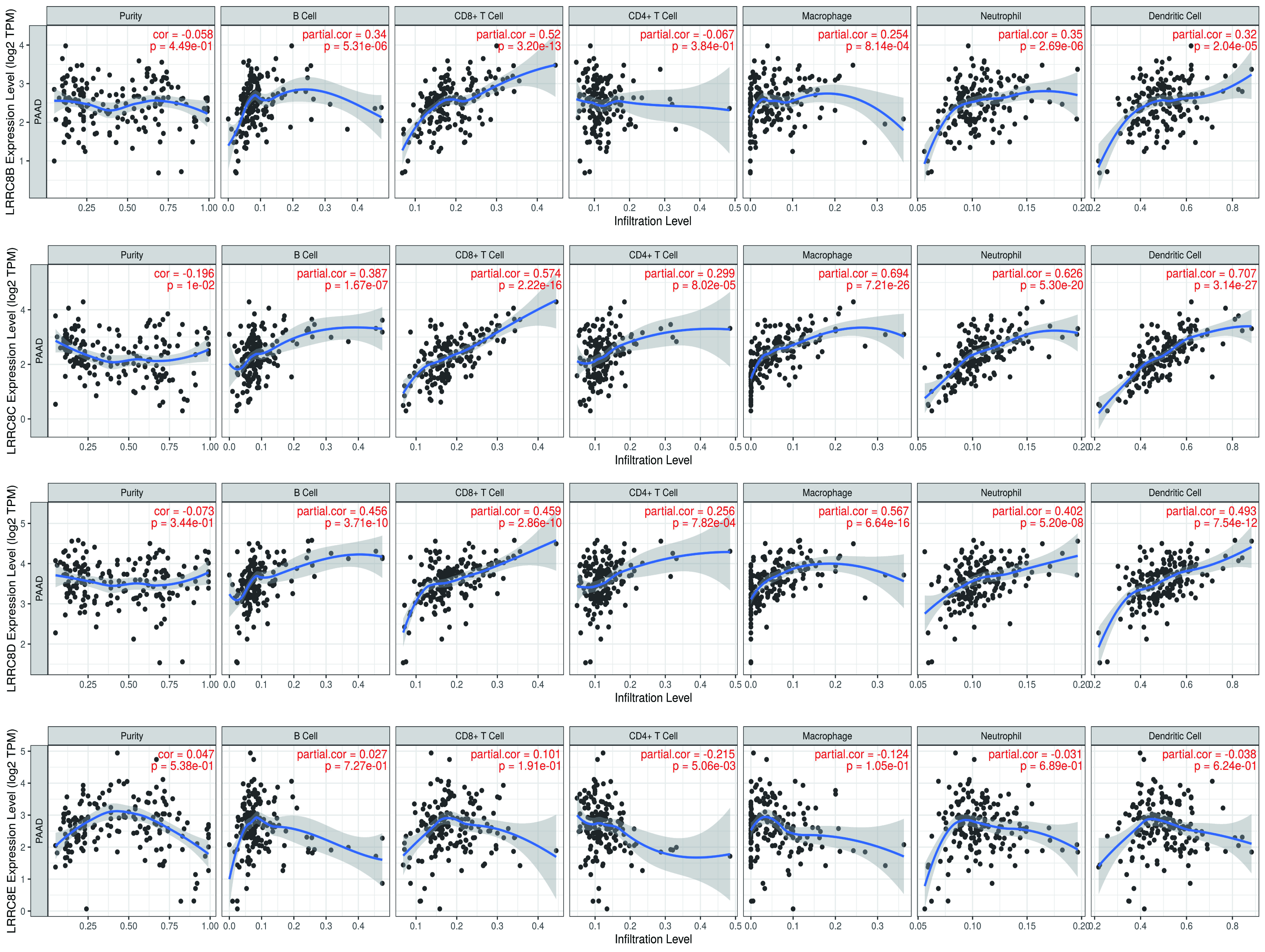

Supplement: Supplementary file 1 [file cancers-14-05526-s001.zip › Figure S2.tif]
