# Supplementary material for: LRRC8A Is a Promising Prognostic Biomarker and Therapeutic Target for Pancreatic Adenocarcinoma
Source: Cancers (Basel). 2022 Nov 10;14(22):5526. doi: 10.3390/cancers14225526 (PMC9688930; doi:10.3390/cancers14225526)

Dear editor,

Thank you for your careful check. I'll present the information you asked in the attachment as follows.

1. For Western blot figures, experiments were conducted 3 times at least, I put the raw pictures of Figure 2(d), 2h and supplementary figure 1d in below. The relative grayscale value of each band relative to actin obtained by using Image J was also showed. The relative protein expression of LRRC8A in PAAD cell lines in Figure 2(d) was based on their relative grayscale value.

   a. **Protein expression of LRRC8A in HPCY5, ASPC-1, PANC1, BXPC-3, ASPC-1^{GEM-R}, ASPC-1^{GEM-R} cells (Left) and protein expression of ACTIN in HPCY5, ASPC-1, PANC1, BXPC-3, ASPC-1^{GEM-R}, ASPC-1^{GEM-R} cells (Right).**

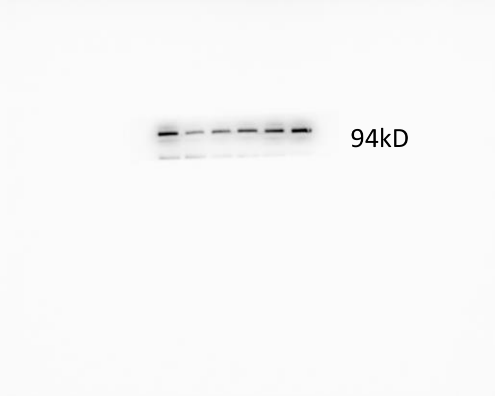

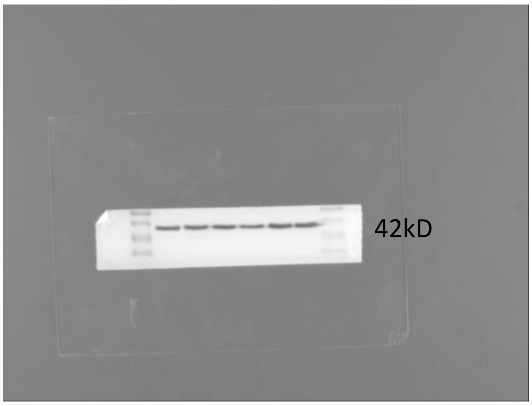

   b. **Protein expression of LRRC8A in HPCY5, , ASPC-1, PANC1, BXPC-3, ASPC-1^{GEM-R} cells (Left) and protein expression of ACTIN in HPCY5, ASPC-1, PANC1, BXPC-3, ASPC-1^{GEM-R} cells (Right).**

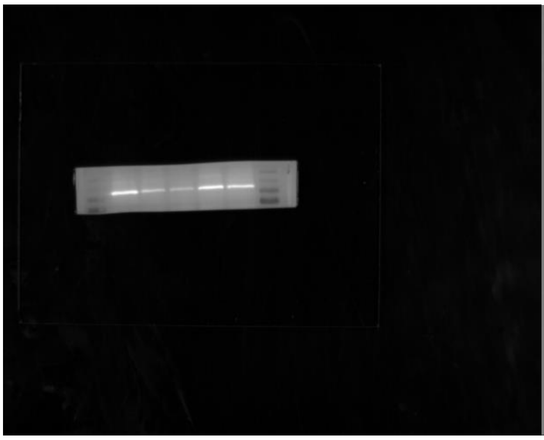
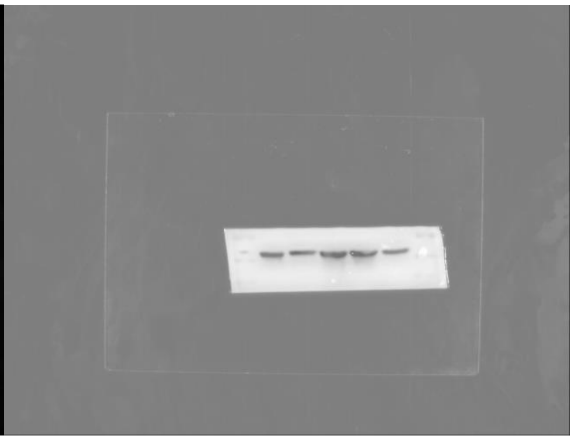

   c. **Protein expression of LRRC8A in ASPC-1 and ASPC-1^{GEM-R} cells (Left) and protein expression of ACTIN in ASPC-1 and ASPC-1^{GEM-R} cells (Right).**

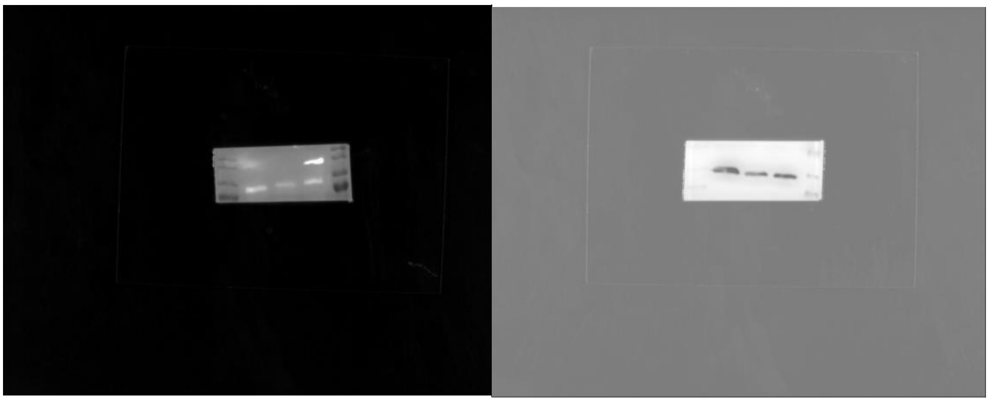

d. **Protein expression of LRRC8A in BXPC-3, ASPC-1 and PANC1 cells and (Left) and protein expression of ACTIN in BXPC-3, ASPC-1 and PANC1 cells.**

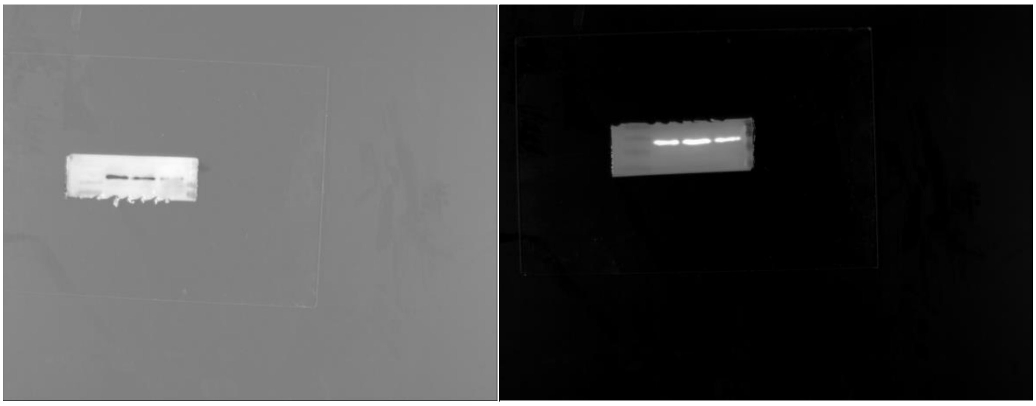

The relative grayscale value of each band (ASPC-1, PANC1, BXPC-3, ASPC-1GEM-R) relative to actin is listed in below.

ASPC1	PANC1	BXPC3	ASPC1^{GEM-R}
0.57652	0.46321	0.97696	0.887790
1.37580	1.02821	2.74920	2.028100

e. **Typical protein expression of LRRC8A in ASPC-1^{GEM-R} cells and ASPC-1^{GEM-R} cells infected with shLRRC8A (left), ASPC-1 cells, ASPC-1 cells infected with shLRRC8A, BXPC-3, BXPC-3 cells infected with shLRRC8A (right) in Figure 2h..**

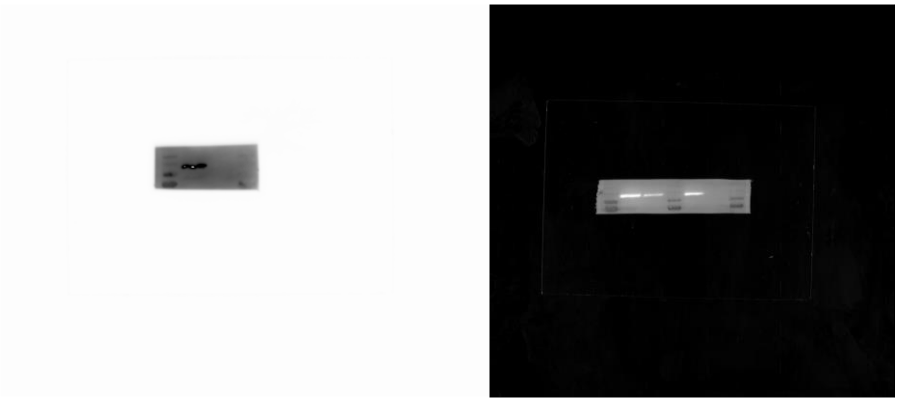

f. **Typical protein expression of MMP2 in ASPC-1^{GEM-R} cells and ASPC-1^{GEM-R} cells infected with shLRRC8A in Figure 2h.**

MMP2: 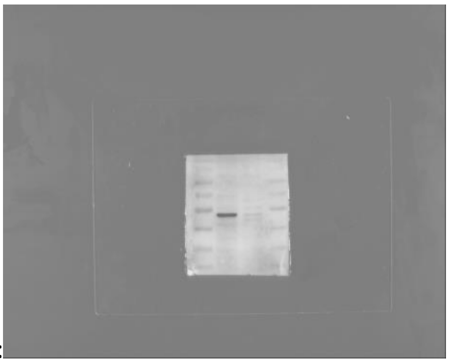

ACTIN: 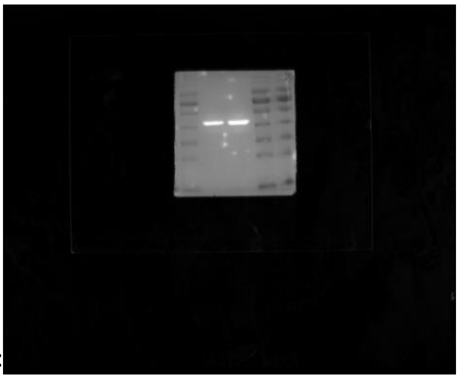

g. **Typical protein expression of MMP2 in ASPC-1 cells and ASPC-1 cells infected with shLRRC8A in Figure 2h.**

MMP2: 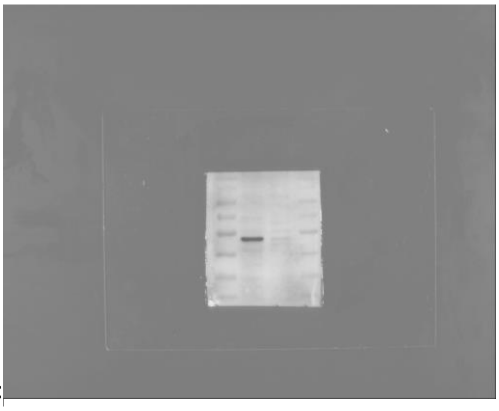

ACTIN: 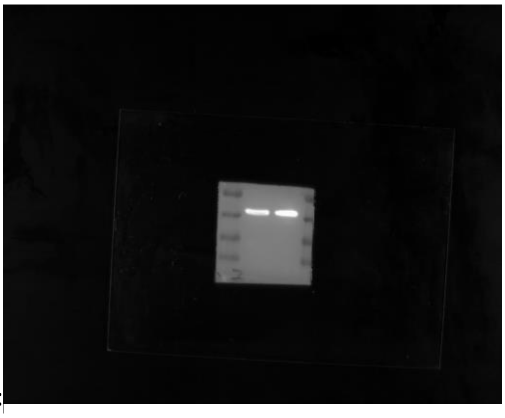

h. **Typical protein expression of MMP2 in BXPC-3 and BXPC-3 cells infected with**

shLRRC8A in Figure 2h.

MMP2: 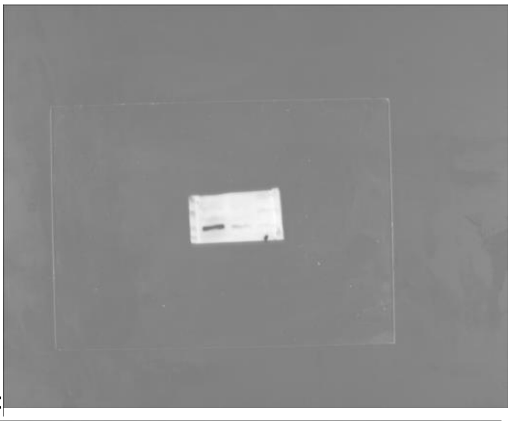

ACTIN: 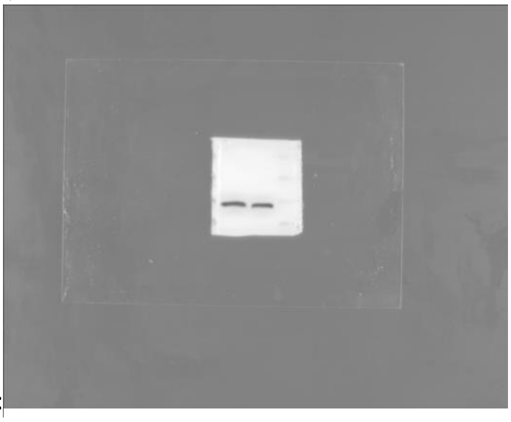

i. **Typical protein expression of MMP2 in ASPC-1^{GEM-R} cells and ASPC-1^{GEM-R} cells treated with GEM or DCPIB+GEM in supplementary figure 1d.**

LRRC8A: 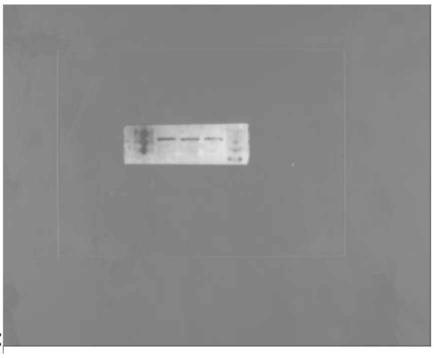

ACTIN: 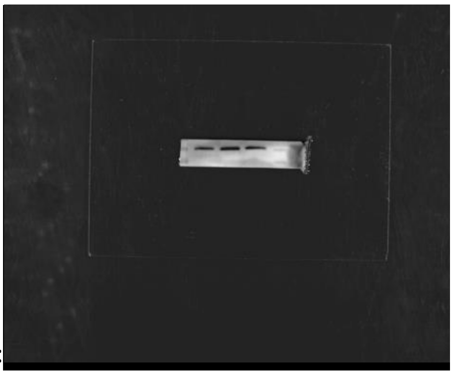

Supplement: Supplementary file 1 [file File S1.pdf › File S1.pdf]
